# Gray matter alteration associated with pain catastrophizing in patients 6 months after lumbar disk surgery: a voxel-based morphometry study

**DOI:** 10.1097/PR9.0000000000000617

**Published:** 2017-08-04

**Authors:** Omar Chehadi, Boris Suchan, Kerstin Konietzny, Odo Köster, Tobias Schmidt-Wilcke, Monika I. Hasenbring

**Affiliations:** a Department of Medical Psychology and Medical Sociology, Faculty of Medicine, Ruhr University of Bochum, Bochum, Germany; b Institute of Cognitive Neuroscience, Department of Clinical Neuropsychology, Ruhr University of Bochum, Bochum, Germany; c Department of Radiology, St. Josef Hospital, University Hospital of Bochum, Bochum, Germany; d Department of Neurology, BG-Hospital Bergmannsheil, University Hospital Bochum, Bochum, Germany

**Keywords:** Pain Catastrophizing, Voxel-Based Morphometry, Magnification, Helplessness, Low back pain

## Abstract

**Introduction:**

Pain catastrophizing (PC), the increased attention to harmful aspects of pain (magnification), and the belief of low controllability (helplessness) has been shown to be important in the maintenance of pain. A growing research on structural alterations in chronic pain revealed an association between PC and brain areas that are involved in affective and cognitive pain processing. However, little is known about a differential effect of the components magnification and helplessness.

**Objective:**

The aim of this study was to identify brain regions associated with overall PC and with the single components of magnification and helplessness.

**Methods:**

We investigated 29 patients 6 months after lumbar disk surgery using voxel-based morphometry. Magnification and helplessness were assessed with subscales of the Avoidance-Endurance Questionnaire (AEQ); overall PC was computed using the sum score. The structural brain imaging data were preprocessed and analyzed using SPM8 and VBM8. Multiple regression analyses were performed with PC scales as predictors and depression as covariate.

**Results:**

Pain catastrophizing was significantly associated with cortical gray matter in brain areas involved in processing attentional, sensory, and affective aspects of pain, including the left posterior cingulate cortex, supplementary motor area, and medial frontal gyrus. In addition, we provide preliminary evidence for brain structure association related to magnification and helplessness.

**Conclusion:**

This study provides evidence for PC-related structural alterations in patients with low back pain, which are consistent with findings from previous research in different chronic pain disorders. This study is the first to evaluate brain changes related to differential domains of PC.

## Abstract 2

Supplemental Digital Content is Available in the Text.

The link between pain catastrophizing and the differential effect of the components magnification and helplessness on brain morphology in patients with low back pain

## 1. Introduction

Pain catastrophizing (PC) has been shown to be an important predictor for pain, pain-related disability, and accompanying emotional distress among patients with chronic pain^19,38,42^ as well as a valid prognostic factor in patients with subacute back pain.^49^ Pain catastrophizing is defined as “an exaggerated negative mental set brought to bear during actual or anticipated painful experience”^42^ comprising at least 3 components: magnification, which reflects increased attention to possible harmful aspects of pain, cognitions of helplessness, reflecting the belief of lacking or ineffective coping strategies and finally rumination, the perseveration of thoughts of magnification and helplessness. Within a broader stress conception, magnification comprises cognitions of primary appraisals (the evaluation of the stimulus as harmful), whereas helplessness refers to secondary appraisal, the belief in coping possibilities and their successfulness.^23^ Although the 3 components are positively related,^7,43^ previous research has shown differential correlations with pain intensity and further outcomes in patients with chronic pain disorders. For example, Sullivan et al.^44^ reported helplessness as a unique predictor for affective pain intensity in patients with neuropathic pain. More recently, in a large scale study on 844 patients with mixed diagnoses of chronic pain, Craner et al.^44^ could demonstrate that helplessness and magnification are unique predictors of several pain outcomes, whereas rumination revealed zero-order correlations that disappeared in the multivariate models. With respect to the fact, that PC and moreover, the respective single components are highly important in the experience of pain, there is a need to better understand the mechanisms underlying PC.

A growing evidence from research in functional neuroimaging has shown that overall PC is associated with higher activation in brain regions that are involved in affective processing of pain, attention to pain, and the cognitive control of emotion and cognition.^33^ Similar findings were reported in studies that examined brain structure alterations in gray matter volume (GMV). For example, Hubbard et al.^16^ reported negative correlation between PC in patients with migraine and GMV in the left primary somatosensory cortex (S1), medial prefrontal cortex (PFC), and anterior midcingulate regions known to be involved in processing the attentional, sensory, and affective aspects of pain and cognitive inhibitory control. Furthermore, PC scores were significantly associated with cortical thickness in supplementary motor area (SMA), MCC and posterior cingulate cortex (PCC).^35^ Because the measure used in previous research assessing PC did not distinguish between components such as magnification or helplessness, it is still unclear whether these single components may be, at least partly, associated with different brain regions. A deeper understanding of the role of PC and their subdomains in pain processing should help to improve treatment outcomes in patients with chronic pain.

In this study, we investigated brain structural alterations associated with overall PC and the components, magnification and helplessness, in a homogeneous sample of patients with respect to their disease history. Keeping in mind that magnification reflects an increased attention and awareness of threat appraisal, we expected brain structure associations in regions known to modulate attention and affective reactions, such as the anterior cingulate cortex (ACC) and anterior insula^16^ and sensory-discriminative aspects of pain somatosensory cortices and PCC.^16^ By contrast, helplessness that mirrors an evaluation of the inability to control pain experiences effectively should be more associated with the brain in regions such as ventrolateral PFC, ACC,^34^ and SMA.^35^

## 2. Materials and methods

### 2.1. Subjects

Subjects were recruited from the St. Josef University Hospital of Bochum (Germany). The sample consisted of 29 individuals (n = 14 female) who participated in the physical examination and had signed the informed consent. The inclusion criteria were as follows: age 18 years and older, a lumbar spine surgery 6 months before the study, and low back pain (LBP; Th 12—L5/S1) during the past 3 months. The exclusion criteria were radiation of pain below the knee, an indication for a second operation because of new acute spinal origin of persistent or recurrent pain, severe osteoporosis, autoinflammatory arthritis, cancer, vertebral fracture, inflammatory disease of the spine, or a previous known psychiatric disease (ie, depression, schizophrenia), and insufficient knowledge of the German language. The study was approved by the ethics committee of Ruhr-University of Bochum and was conducted in accordance with the Declaration of Helsinki.

### 2.2. Behavioral and clinical measures

Each participant completed a series of self-report questionnaires to measure the following parameters:

*Pain Catastrophizing* was assessed with 2 of the cognitive subscales, scales of the German version of the Avoidance-Endurance Questionnaire (AEQ).^14^ Cognitive responses were defined as automatically occurring thoughts, introduced by the question “When I become aware of my pain during the past 14 days, this thought comes to my mind….” The AEQ contains the components of magnification (Catastrophizing Thoughts Subscale, CTS) and helplessness (Help/Hopelessness Scale, HHS). Both scales have shown moderate to high positive correlations,^13,14^ reflecting overlapping but also distinct variation. The reliability, as indicated by internal consistency, for the overall CTS score in the current sample was 0.96 (Cronbach alpha).

*Magnification* was assessed with the 3-item scale CTS of the AEQ describing the exaggeration of the possible seriousness of pain sensations (eg, “I can't have a tumor, can I?” and “It isn't a serious illness, is it?”). The items were answered on a 7-point numerical scale that ranges from “never” to “always.” Higher scores indicate a higher tendency of magnification. The CTS has been shown reliable, indicated by internal consistencies between 0.78 and 0.84^13,14^ and valid with respect to low but positive correlations with pain and disability and moderate to high positive associations with pain anxiety and fear-avoidance beliefs.^14^

*Helplessness* was assessed with the 9-item scale HHS of the AEQ assessing automatic cognitions reflecting low effectiveness and low controllability of pain (eg, “Whatever will I do if it gets worse again?” “Nothing helps anymore!”). All 9 items are answered on a 7-point numerical scale that ranges from “never” to “always.” Higher scores indicate more frequent thoughts of helplessness. The HHS has been shown high internal consistency (Cronbach alpha 0.91,^14^), and positive correlations with pain, disability, and pain-related affective distortion such as pain anxiety and fear of pain-related (re)injury.^14,36^

#### 2.2.1. Depressive mood

We used the German version of the 21-item Beck Depression Inventory (BDI) (German version Kammer 1983).^3,18^ The BDI is a self-administered questionnaire that assesses the severity of depressive symptoms. Internal consistency assessments of reliability have been high with Cronbach alpha above 0.90 in most evaluations.^2^ The sum score of the BDI ranges from 0 to 63; higher scores indicate a higher degree of depressive mood.

#### 2.2.2. Pain variables

Low back pain disease characteristics including self-reported measures of disease duration (months) and ratings of average pain intensity using a 0 to 10 numerical rating scale (NRS; 0, no pain and 10, worst pain imaginable) for the past week and past 3 months were determined.

### 2.3. Scanning protocol and data processing

A structural high-resolution scan of each participant was acquired using a T1-weighted MPRAGE sequence (Field Of View (FoV): FoV: 250 mm, echo time = 4.18, repetition time = 1990 ms) on a 1.5 T, Siemens Symphony scanner. One hundred sixty sagittal slices with a resolution of 1 × 1 × 1.5 mm were recorded.

Analysis was performed using the SPM8 package (Wellcome Department of Imaging Neuroscience, London, United Kingdom; http://www.fil.ion.ucl.ac.uk/spm) in combination with Matlab 7.9 platform (Mathworks, Sherborn, MA).

Preprocessing has been performed using VBM8 (VBM8 toolbox, download from http://dbm.neuro.uni-jena.de/vbm8/) with default parameters (bias regularization 0.0001 and bias cutoff full width at half maximum 60 mm). Briefly, anatomical images were spatially normalized into the Montreal Neurological Institute space and segmented into gray matter (GM), white matter, and cerebrospinal fluid. Each GM segment was morphed into a customized DARTEL template, which was in stereotactic space of the Montreal Neurological Institute. Finally, the modulated GM segments were smoothed with a Gaussian kernel set at 8-mm full width at half maximum.

### 2.4. Statistical analysis

The smoothed GM images were entered into a voxel-wise multiple regression analysis to investigate the variability in the regional GMV. We excluded all voxels with GM values of <0.1 using absolute threshold masking. Statistical analyses were performed using the general linear model in SPM12. In addition, we used AlphaSim^48^ to calculate the appropriate, corrected for multiple comparison threshold, cluster size for the whole-brain analysis. 5000 Monte Carlo simulations using AlphaSim with a voxel-level threshold of *P* < 0.001 were calculated resulting in a cluster of 44 contiguous voxels reflecting a corrected threshold of *P* < 0.05. Previous studies suggest that total GMV^31^ and age^17,31^ are important covariates in VBM. Therefore, both covariates were included in all the following analyses: first, the correlation between the GMV and overall score of PC; second, the correlation between the GMV and the mean of pain magnification; and finally, the correlation between the GMV and the mean of helplessness. In addition, previous studies have reported that depression was highly correlated with PC, and there is also a debate whether catastrophizing is a symptom of depression or a separate construct.^10^ Therefore, we reran the models including depression as a covariate to control that our results were robust with respect to depression symptoms. Anatomical labeling of brain regions showing significant correlations and the visualization of results was performed using the SPM8 extension XjView (http://www.alivelearn.net/xjview8/) and the Wake Forest University (WFU) (http://fmri.wfubmc.edu/software/pickatlas).^21,22,26^

In addition, we tested whether the observed differences in the height of correlation between GMV and overall PC (PC-sum score), magnification, and helplessness are significant, using the Pearson and Filon z-score,^8^ which is part of the cocor package (http://comparingcorrelations.org/). The cocor package covers a broad range of tests, including the comparisons of dependent correlations with either overlapping or nonoverlapping variables.

## 3. Results

With respect to the behavioral data, we found a positive correlation between overall PC and the BDI (*r* = 0.468, *P* = 0.01) and average pain intensity last week (*r* = 0.437, *P* = 0.018). In addition, helplessness was significantly positively correlated with BDI (*r* = 0.588, *P* = 0.001) and with pain intensity (*r* = 0.435, *P* = 0.018) but not with magnification, assessed with the CTS (*r* = 0.275, *P* = 0.149). Furthermore, magnification was not significantly correlated with BDI (*r* = 0.082, *P* = 0.672) and with pain intensity (*r* = 0.237, *P* = 0.216). In addition, no significant correlation was found between magnification and pain duration (*r* = −0.274; *P* = 0.151) and between helplessness and pain duration (*r* = 0.170; *P* = 0.377). Table 1 reports mean values and SDs for all descriptive variables.

**Table 1:**
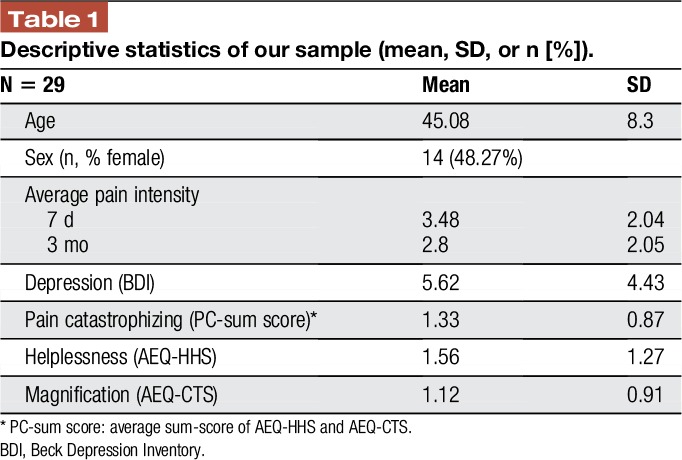
Descriptive statistics of our sample (mean, SD, or n [**%**]).

### 3.1. Imaging results

Imaging results showed positive relations between overall PC and GMV in the right MFG/SMA which remained robust, when depression was regressed out. By contrast, a negative correlation between overall PC and GMV in the left PCC merely appeared when we controlled for depression.

A negative correlation between GMV in the left PCC was further seen for magnification, but not for helplessness, irrespective of the control for depression. Furthermore, magnification revealed a positive correlation with GMV in the left MFG, also irrespective of depression.

**Figure 1.**
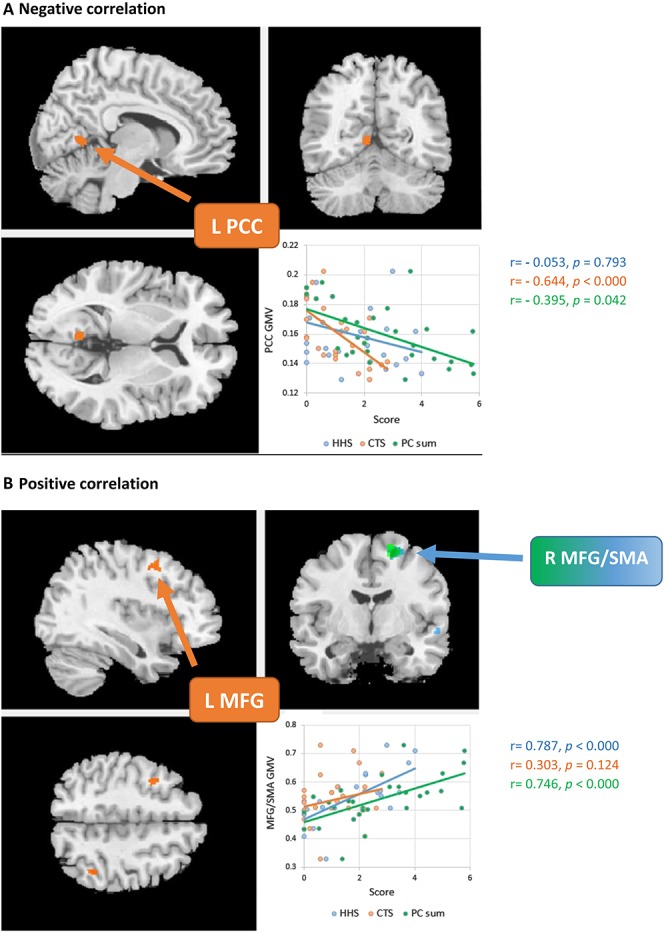
Correlations between pain catastrophizing (PC-sum score), the subscales helplessness (AEQ-HHS) and magnification (AEQ-CTS) and regional gray matter volume. Part A shows that there is a negative correlation between GMV in PCC and magnification, whereas part B shows positive correlation between GMV in MFG/SMA and helplessness. Statistical maps were set at a cluster threshold of 44 voxels corresponding to a corrected *P* < 0.05, (corrected for multiple comparisons using AlphaSim Monte Carlo simulations). GMV, gray matter volume; MFG, medial frontal gyrus; PCC, posterior cingulate; SMA, supplementary motor area.

With respect to helplessness, we found a positive correlation with GMV in the right MFG/SMA, which also remained robust for depression. To note, this positive association was seen for the overall score of PC, even irrespective of depression. No significant correlations were identified in the ACC and PFC. For details (ie, peak coordinates and z values) see Table 2 and Figure 1. In addition, we found no significant correlation between total GMV and pain duration (*r* = −0.098; *P* = 0.605). Furthermore, pain duration did not correlate significantly with GMV findings in MFG and PCC. For a complete exploratory result, see the Supplementary Data (Supplementary Table 1, Supplementary Table 2; available online at http://links.lww.com/PR9/A9 and Supplementary Figure 1; http://links.lww.com/PR9/A8).

**Table 2:**
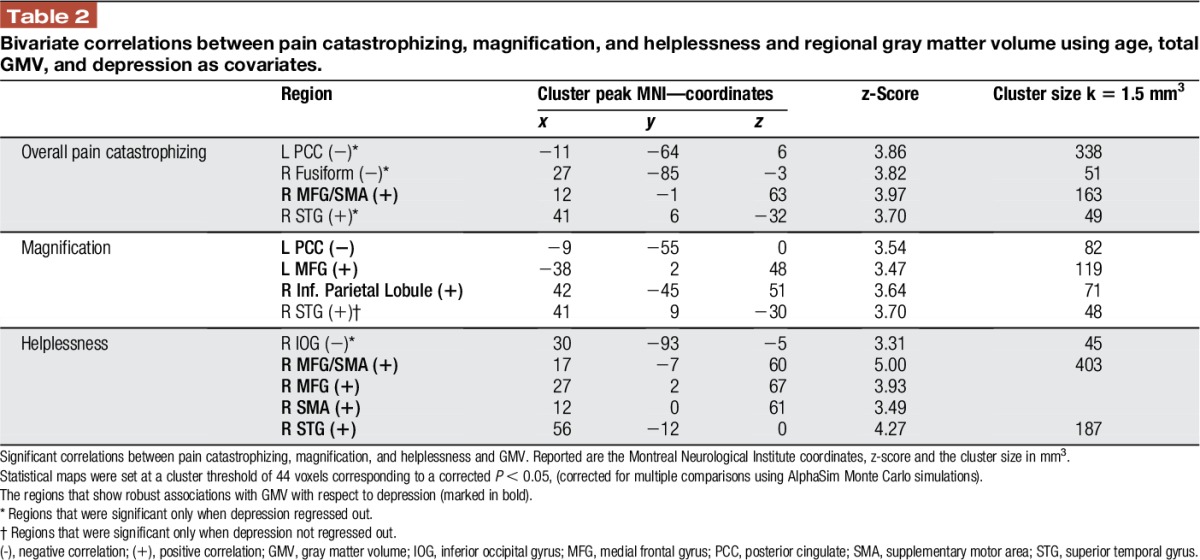
Bivariate correlations between pain catastrophizing, magnification, and helplessness and regional gray matter volume using age, total GMV, and depression as covariates.

When we compared the correlation between regional GMV and all 3 components of PC, we found significant differences in the correlation between GMV in PCC and magnification and helplessness, respectively (*z* = −2.751, *P* = 0.003) as well as between GMV in PCC and magnification and overall PC, respectively (*z* = −1.985, *P* = 0.024). Although both, the correlation between GMV in PCC and magnification as well as overall PC are significant and with a negative direction, the correlation for magnification was significantly higher than for overall PC. In addition, we found significant differences in the correlation between GMV in the right MFG/SMA and helplessness and magnification, respectively (*z* = 2.77, *P* = 0.003) but not between GMV in the right MFG/SMA and helplessness and overall PC, respectively (*z* = 0.613, *P* = 0.27). Finally, we found significant differences in the correlation between GMV in the left MFG and helplessness and magnification, respectively (*z* = 2.0217, *P*-value = 0.0432). For details (ie, r and *P* values) see Figure 1.

## 4. Discussion

As hypothesized, overall PC as well as both subcomponents of magnification and helplessness were significantly associated with cortical GM in brain areas involved in processing attentional, sensory, and affective aspects of pain, including the left PCC, STG, and MFG/SMA. In addition, the behavioural results of the current study are in line with previous studies investigating the different aspects of PC in patients with subacute and chronic pain.^7,14,36,42^ These data support the idea that PC is a multifaceted construct comprising pain-related cognitions such as magnification and helplessness which share overlapping as well as distinct parts of variance and are differently related to pain outcomes. To our knowledge, no study to date has examined brain structure association related to overall PC and the components of helplessness and magnification in patients with LBP.

### 4.1. Brain regions associated with overall pain catastrophizing

Our VBM analysis yields evidence that overall PC was significantly positively associated with GMV in the right MFG that is known to play a role in processing of negative emotion.^29^ The association in this region was robust with respect to depression. Gray matter volume in this area was also positively correlated with helplessness but not with magnification. When we compared the height of these correlations, we found no significant differences between helplessness and overall PC. Comparable results were found in previous research that reported positive correlation between SMA and overall PC.^35^ However, the authors reported that this correlation was no longer significant after control for helplessness. This supports the idea that the right MFG/SMA is merely associated with helplessness but not with overall PC.

Of interest, negative correlations between overall PC and GMV were only found when depression was used as an extra regressor. This raises the possibility that any observed negative correlations with overall PC are actually a function of removing depression. However, we found a negative association between overall PC and GMV in the PCC; this region is known to be a central part of the default mode network^6^ and commonly activated during processing of painful as well as emotional stimuli. In addition, our results are in line with findings from previous functional imaging studies in reporting positive correlations between individual PC scores and PCC activation.^37^ In addition, GMV in the left PCC has been reported to be negatively correlated with migraine pain intensity.^16^ Patients suffering from migraine headaches are more likely to display higher levels of PC compared with migraine-free individuals.^15,16^ Our results in this study are in line with earlier studies that reported structural abnormalities in several brain regions in patients with chronic pain, eg, the GMV of patients with chronic back pain was reduced in the dorsolateral PFC and the right thalamus,^1,39^ in the ACC and insula^27,28^ as well as in the PCC compared with healthy controls.^5^ However, only 1 study reported correlations between PC and reduced cortical GM in the dorsolateral PFC, medial PFC, inferior frontal gyrus, middle temporal gyrus, and S1.^16^ These discrepant findings may be attributed to the fact that the authors investigated PC in patients with migraine disease and that in some diagnoses, disease variables may play a more important role in the relation between PC and brain abnormalities.^32^

### 4.2. Brain regions associated with magnification

One important goal of our study was to investigate the association between GMV and the single components of PC, magnification, and helplessness. Concerning magnification, we found a strong association with GMV in the left PCC. To note, GMV in the left PCC was merely associated with overall PC (when depression was regressed out) and with magnification, but not with helplessness. The negative correlation between PCC and magnification was also robust after control for depression. These findings are in accordance with findings from functional imaging studies showing a strong relationship between PC and an activation in the PCC in patients with LBP and fibromyalgia.^10,24^ In addition, the PCC plays a prominent role in the processing of emotionally salient stimuli,^25^ and activation of the PCC is correlated with severity of anxiety symptoms in mood and anxiety disorders.^4^ However, further evidence suggests that the PCC plays an important role in regulating the focus of attention^11^ and that it is involved in orienting the body towards innocuous and noxious somatosensory stimuli.^47^ As Sullivan and colleagues proposed,^41^ we may conceptualize PC as automatic cognitions in the sense of primary and secondary appraisal sensu Lazarus and Folkman (1984).^23^ Primary appraisal involves attending to and evaluating the meaning of a situation, whereas secondary appraisal refers to an evaluation of possible coping strategies and their effectiveness or controllability. Gray matter volume association with the PCC may provide a possible explanation for the behavioral data of previous studies reporting that high catastrophizing individuals show increased attention to pain and a certain inability to divert attention from pain.^40,46^

### 4.3. Brain regions associated with helplessness

Concerning helplessness, our results revealed a significant positive association between GMV in the right MFG/SMA and helplessness. These findings are consistent with previous structural magnetic resonance imaging research demonstrating a positive correlation between helplessness and cortical thickness in this region.^35^ In addition, our results are in line with previous research showing that right MFG is involved in negative emotions such as fearful faces processing^9^ and the imagination of shame-related situations.^29^ It may be speculated that experiencing helplessness and hopelessness when trying to cope with pain may elicit or increase feelings of shame. Negative emotion in general in patients with chronic pain was significantly positively correlated with PC.^30^ Lateralization of GMV with respect to the subdomains of PC is of further interest. Although helplessness displayed associations with the right MFG/SMA, magnification revealed a positive relation with the left MFG. Talati and Hirsch^45^ reported effects of lateralization in the MFG with respect to decision making and executive functions, although the relation to catastrophizing and its subdomains remains unclear. Further research is warranted to explore possible effects of lateralization and subdomains of PC.

This study might have some limitations. The design is cross sectional; hence, it is not possible to determine whether the relationship between GMV alterations and PC is causal and, if so, to determine the direction of the relationship between pain psychology and brain structure. An additional limitation of our study is the absence of a control group. It remains unclear whether the GMV associations identified in our study are specific to PC in CLBP or exist in the general population. However, studies without control group can also be helpful for identifying trends that can be studied more in-depth. Thus, longitudinal studies will be important to fully confirm the hypothesis that PC can alter GMV. In addition, VBM is very helpful in detecting macrochanges in regional brain morphology; the correlating changes in cytoarchitecture remain to be fully elucidated.

Further limitation should be considered regarding the overall score for PC. Helplessness and magnification were positively but not significantly correlated in our sample, although moderate to high correlations were reported for these scales in previous research.^13,14^ Low to moderate correlations between the subcomponents of PC were further reported by the validation study of the Pain Catastrophizing Scale (PCS),^43^ one of the most used measures of magnification and helplessness. With respect to the high internal consistency of the overall PCS score, the authors concluded that the subcomponents “can be viewed as different dimensions of the same construct.”^43^ Thus, the impact different aspects of PC, such as magnification and helplessness, will have on different pain outcomes^7^ or on the activity of distinguishable brain circuits remains an important research topic that should be addressed in future studies.

Another limitation is that we did not include a measure for rumination, a further aspect of PC.^43^ The role of rumination, the perseveration of negative thoughts on causes, and consequences of pain in the maintenance of pain and disability is still under debate.^7^ However, Kucyi et al.^20^ reported data on an enhanced medial prefrontal-default mode network due to pain rumination in patients with chronic pain. Thus, further research on the role of pain rumination in structural brain alterations in patients with chronic pain disorders is warranted.

However, an important strength of this study is the homogeneity of our sample regarding the disease history: All subjects underwent a first lumbar disk surgery 6 months before the study. We excluded patients who suffered from pain radiating below the knee or showed signs indicating the necessity of a second operation besides other possible specific causes of LBP (ie, an inflammatory disease of the spine).^12^ Selecting a homogeneous patient sample regarding the disease history is important because different subpopulations may have diverse coping strategies that can influence their pain outcomes. For example, pain conditions with a neuropathic etiology are often distinct from other painful conditions without damage to the nervous system.^39^ A further strength of our study is the control for potential confounders, as catastrophizing is known to be strongly associated with depressive symptoms; the possible influence of depression was statistically removed.^10^ However, given the considerable conceptual overlap between depression and helplessness (negative cognitions) and the high correlation between them, including depression as a covariate may change the results of helplessness far more than magnification. Therefore, we reran the analyses without depression as a covariate to confirm that our results are not a function of controlling for depression.

## 5. Conclusion

Our results indicate that in a sample of patients with LBP 6 months after a primary lumbar spine surgery, GM brain associations with regions involved in the affective and cognitive processing of pain vary with respect to overall PC, whereas the subcomponents of magnification and helplessness seem to share some variance but also display unique parts in the association with these brain regions. Even the behavioural data indicated that both domains of PC are differentially related to pain and depression. These findings may help us to better understand the neuronal correlates of PC with the components of more attention-related aspects of magnification and helplessness, which reflects inefficiency of coping and perceived uncontrollability of pain.

## Disclosures

The authors have no conflicts of interest to declare.

## Appendix A. Supplemental Digital Content

Supplemental digital content associated with this article can be found online at http://links.lww.com/PR9/A9 and http://links.lww.com/PR9/A8.
